# Superior Ophthalmic Vein Thrombosis in Trinidad and Tobago: A Case Series

**DOI:** 10.7759/cureus.72900

**Published:** 2024-11-02

**Authors:** Kieron Naguar, Steven Sankar, Anala A Maharaj, Jeanmarc Sookhoo, Ryan R Ramoutar

**Affiliations:** 1 Ophthalmology, San Fernando General Hospital, San Fernando, TTO; 2 Radiology, San Fernando General Hospital, San Fernando, TTO; 3 Ophthalmology, University Hospitals of Leicester NHS Trust, Leicester, GBR

**Keywords:** facial cellulitis, idiopathic orbital inflammatory disease, ophthalmology, orbital cellulitis, paranasal cellulitis, proptosis, superior ophthalmic vein thrombosis, thyroid eye disease, trinidad, trinidad and tobago

## Abstract

Superior ophthalmic vein thrombosis (SOVT) is a rare and potentially sight and life-threatening condition. Although broadly classified into septic and aseptic causes, its etiology is widely varied and presents clinically with a range of signs and symptoms, leading to diagnostic and therapeutic challenges. This case series describes three cases of radiologically confirmed SOVT, presenting in close succession at an ophthalmology department in a single center in Trinidad and Tobago. All three cases were managed with a multidisciplinary approach and ultimately demonstrated full clinical resolution.

## Introduction

Superior ophthalmic vein thrombosis (SOVT) is a rare but potentially serious condition characterized by the formation of a thrombus within the superior ophthalmic vein (SOV) [1]. Due to its rarity, the exact incidence of SOVT still remains unclear, however, a recent case series has quoted its incidence as 3-4 cases/million/year [1].

SOVT typically presents with a constellation of symptoms, including lid edema, periorbital pain, chemosis, hyperemia, dilated and tortuous episcleral and conjunctival vessels, proptosis, ophthalmoplegia, and optic neuropathy [2,3]. These symptoms are all subsequent to impaired venous output from the orbit. 

The etiology of SOVT varies widely but can be broadly categorized into septic and aseptic causes [4]. Aseptic causes include autoimmune or systemic conditions, trauma, hematological conditions, malignancies, vascular malformations, and hormonal therapy [1,2]. Septic causes include orbital infections, facial infections, sinusitis, and dental infections [1,2]. Of note, recent studies have reported cases of SOVT as a result of both COVID-19 infection and the ChAdOx1-S COVID-19 vaccine [5-12].

The clinical manifestations of SOVT can vary widely and these symptoms can mimic a host of differential diagnoses, leading to diagnostic challenges and delays in appropriate management. As such, prompt recognition and management are essential to prevent serious complications such as permanent impairment or loss of vision and death [1-4]. Radiological confirmation plays a critical role in diagnosing SOVT. The radiological investigations of choice are contrast-enhanced CT or MRI [2,13].

This case series highlights three cases of SOVT of varied etiologies all of which presented between April and May 2024 at one center in Trinidad and Tobago. These cases were all confirmed radiologically and successfully managed by a multidisciplinary team until complete resolution.

Methodology

Patients were included in this case series on the basis of radiological confirmation of an SOVT. This was defined as the enlargement of the SOV, containing a filling defect on post-contrast images. For each case, the patient’s demographic data, medical comorbidities, presenting symptoms, treatment, and final outcome were reviewed and documented.

To compare the findings of these cases to those in existing literature, a literature search was conducted on articles indexed on Cureus, PubMed, Google Scholar, and Cochrane Review search engines. Keywords used in the search included ‘ophthalmology’, ‘superior ophthalmic vein’, ‘thrombosis’, ‘paranasal cellulitis’, ‘facial cellulitis,’ ‘orbital cellulitis’, ‘thyroid eye disease’, ‘idiopathic orbital inflammatory disease’, ‘Trinidad’, Trinidad and Tobago’, 'Caribbean' and 'Latin America'. These keywords were used as single search terms or in combination. 

Inclusion Criteria for Literature Review

The inclusion criteria for the literature review were as follows: papers that mentioned SOVT, were fully accessible, written in English, and peer-reviewed.

Articles were not filtered based on the date of publication in order to capture as wide a net as possible for this rare condition. No articles were found to meet the combined search criteria, indicating that this, to the best of our knowledge, is the first case series to describe the findings of SOVT in the patient population in Trinidad and Tobago, the Caribbean, and the wider Latin American geographical region.

## Case presentation

The three subjects of this case series presented within 30 days of each other, at one centre in Trinidad and Tobago. They were all assessed, investigated, and admitted for management. Visual acuity was performed using a Snellen chart. Intraocular pressure measurements were performed by Goldman Applanation Tonometry. Radioimaging was performed using CT and MRI imaging, as detailed below. Please note that any asymmetry in radiological images is due to mild patient rotation during imaging.

SOVT Case 1: Paranasal cellulitis (septic)

A 44-year-old poorly compliant diabetic male presented with a two-day history of left-sided facial swelling. He exhibited left-sided periorbital edema and nasal bridge swelling. His visual acuity was 20/15 in each eye. Intraocular pressures (IOP) measured as follows: right eye, 12mmHg, and left eye, 14mmHg. The rest of his ophthalmic examination was unremarkable. Initial CT scan radiography revealed left paranasal sinusitis with associated preseptal cellulitis. He was admitted under otolaryngology and intravenous antibiotics and analgesia were commenced. His diabetic control was found to be poor, with an HbA1c of 10.7%, for which he was referred to the internal medicine team. 

**Figure 1:**
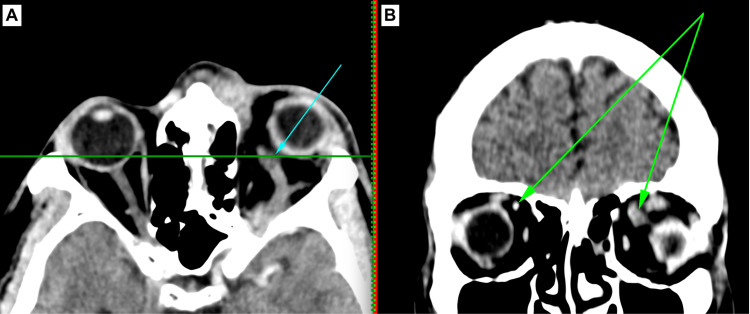
Case 1. Axial contrast-enhanced CT imaging in panel A illustrates a torturous superior ophthalmic vein (blue arrow). The coronal image in panel B displays the normal-appearing contralateral right versus left superior ophthalmic veins (green arrows). Additionally, the axial image in panel A demonstrates thickening of the septum with enhancement of the extraconal fat, medially indicative of pre-septal cellulitis.

While hospitalized, on Day 4 of admission, he complained of new-onset diplopia and increasing left-sided eye pain. He was re-referred to ophthalmology. Ophthalmic examination revealed left-sided lid edema, erythema, chemosis, and dilated and tortuous conjunctival vessels. The left eye displayed non-axial, non-pulsatile proptosis. Hertel's exophthalmometry recorded 21mm in the right eye and proptosis of 25 mm in the left eye. His extraocular movements were limited in all directions of gaze in the left eye. His visual acuity was 20/15 in the right eye and 20/25 in the left eye. IOPs were normal bilaterally. Pupillary reactions and fundal examination were normal bilaterally. A repeated CT scan demonstrated ongoing left paranasal sinusitis, preseptal cellulitis, and a dilated and tortuous left superior ophthalmic vein when compared to the previous scan (Figure 1).

**Figure 2:**
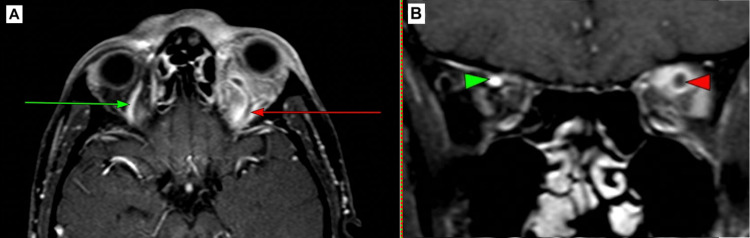
Case 1. Axial contrast MRI-venogram T2-weighted-fat suppressed image in panel A illustrates a left dilated superior ophthalmic vein (red arrow) with a hypointense intraluminal defect indicative of thrombus. This is shown in comparison to the coronal image in panel B which displays the normal-appearing contralateral right superior ophthalmic vein (green arrowhead). The MRI also depicts inflammatory involvement of the left superior and lateral recti muscles with mild changes noted within the intra-conal fat.

A subsequent MRA/MRV was requested which confirmed the diagnosis of a left SOVT with an intraluminal thrombus (Figure 2). In addition to intravenous antibiotics, intravenous dexamethasone 8 mg was added to his therapeutic regimen by the otolaryngology team for the duration of his stay (10 days). Systemic steroids are widely used in the presence of a diagnosis of sinusitis. Internal medicine and hematology were asked for their input in this case. He was subsequently started on enoxaparin (low molecular weight heparin) 100 units subcutaneously, once daily for three months, which was the anticoagulant of choice by the multidisciplinary team due to its ease of reversibility with protamine sulphate. His signs and symptoms improved on this treatment plan and he made a full recovery with full restoration of extraocular movement, resolution of his anterior segment findings and resolved proptosis. He was discharged on Day 10 post-admission with planned follow-up in the hematology and internal medicine outpatient clinics.

SOVT Case 2: Thyroid eye disease (aseptic)

A 46-year-old female presented with a two-week history of painful and progressive left-sided proptosis with accompanying blurred vision. She gave a two-week history of intermittent retro-orbital pain and limitation of abduction. Her general health was otherwise good with no preexisting comorbidities. She was on no regular medications.

Ocular examination revealed left-sided periorbital swelling with erythema, non-pulsatile proptosis, and significant conjunctival chemosis with dilated episcleral vessels. Visual acuity measured as follows: right eye, 20/20; left eye, 20/25. IOPs were recorded as follows: right eye, 16mmHg; left eye, 20mmHg. Dilated slit-lamp fundal examination showed left papillitis which was suspected to be a result of a compressive optic neuropathy. A mild left eye relative afferent pupillary defect was noted. Ocular motility of the left eye was limited in abduction.

**Figure 3:**
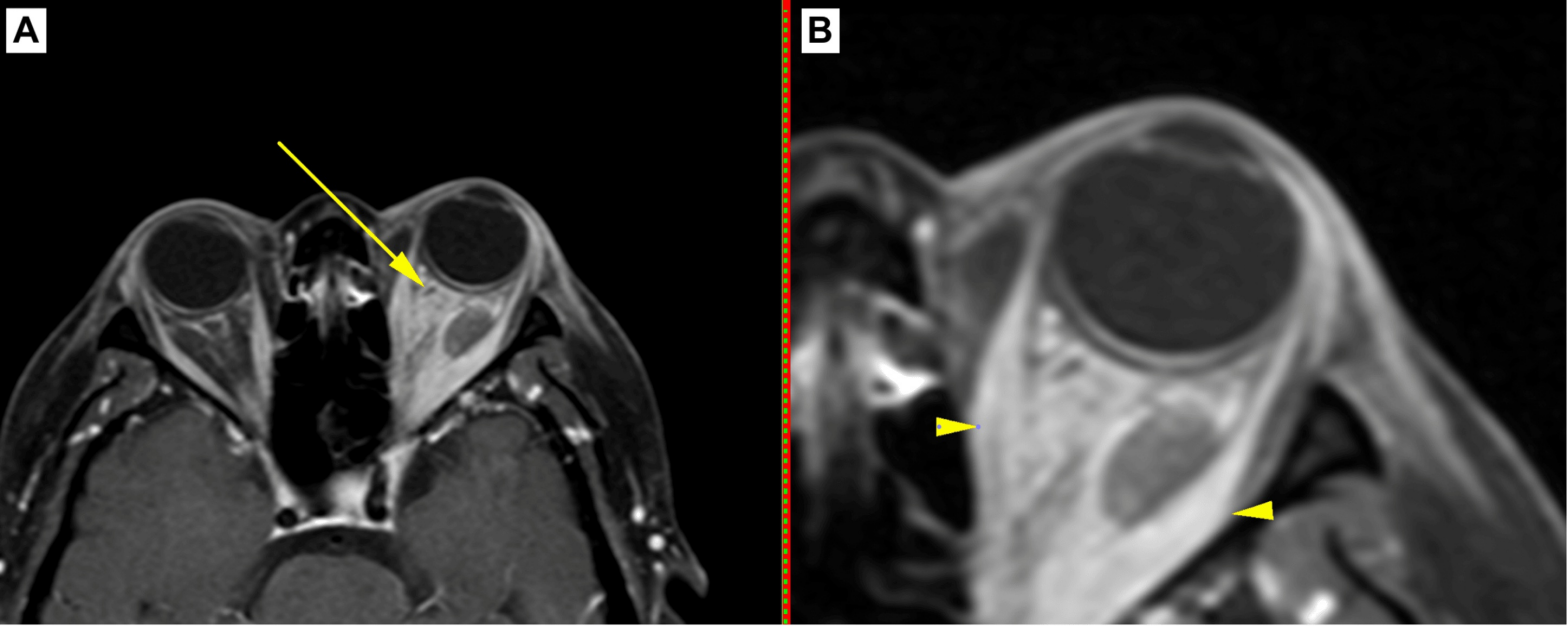
Case 2. Axial contrast MRI-venogram T2-weighted-fat suppressed image in panel A illustrating the left superior ophthalmic vein (yellow arrow) with a subtle hypo-intense intraluminal defect indicative of thrombus. The image in panel B is a focal, more detailed view indicating the thickening of the bellies of the medial and lateral recti muscles (yellow arrowhead) with sparing of the anterior tendons; radiological signs associated with thyroid orbitopathy. Inflammatory changes are also visualized in the congested intraconal fat.

A CT radiograph of the orbits demonstrated left intraconal soft tissue densities. An MR venography demonstrated thickening of the bellies of the left medial and lateral recti muscles with the most prominent thickening noted to be that of the belly of the left lateral rectus muscle. Edema of the retro-orbital and intraconal fat was also noted. A dilated superior ophthalmic vein was noted with an intraluminal thrombus. Cavernous sinus thrombosis was absent (Figure 3).

The radiological findings were suspicious for thyroid eye disease and thyroid function tests (thyroxine (T4), triiodothyronine (T3), and thyroid stimulating hormone (TSH)) were requested. These were normal. Further investigation for thyroid auto-antibodies was indicated; however, the facilities for these tests were unavailable in the hospital setting and the personal cost to the patient to have this test done privately was prohibitive. Further investigations included serum angiotensin-converting enzyme, antineutrophil cytoplasmic antibody, rheumatoid factor, antinuclear antibody, antimicrosomal antibody, and inflammatory markers. These were all negative or within the normal range. A chest X-ray and abdominal ultrasound were also normal.

A diagnosis of SOVT with suspected thyroid eye disease was made based on radiological findings. Idiopathic orbital inflammatory disease was considered as another differential diagnosis. The patient declined an offer of an orbital biopsy which could have resulted in a definitive diagnosis or excluded other disease processes such as orbital lymphoma. Treatment with high-dose oral steroids (prednisolone 80mg once daily) was initiated. After three weeks of treatment, her proptosis resolved, ocular motility had returned to normal, and visual acuity measured 20/20 in the left eye. She underwent a three-month period of slowly tapered steroid treatment and has ongoing outpatient follow-up in the endocrine clinic.

SOVT Case 3: Incidental 

An 84-year-old well-controlled diabetic and hypertensive female presented with a four-week history of progressively worsening right eye pain and swelling. At presentation, she displayed right-sided lid edema, chemosis, axial proptosis, and ophthalmoplegia, worse in infraduction. Her visual acuity was as follows: right eye, 20/100; left eye: 20/40. IOP was elevated in the right eye at 32mmHg and normal in the left eye at 12mmHg. Fundus examination of the right eye revealed optic disc edema with associated flame hemorrhages consistent with compressive optic neuropathy. A mild right eye relative afferent pupillary defect was noted. The left eye examination was normal.

**Figure 4:**
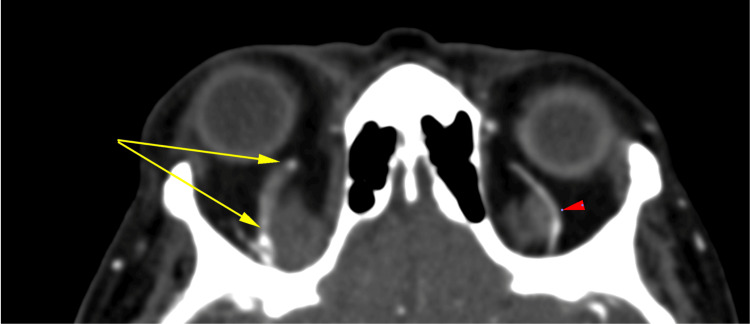
Case 3. Contrast-enhanced CT venogram illustrating a filling defect within the dilated superior ophthalmic vein (yellow arrows) on the right compared to the normal-appearing superior ophthalmic vein on the left (red arrowhead).

An orbital CT scan with contrast revealed right-sided proptosis and a right SOV filling defect, consistent with a SOVT (Figure 4). In collaboration with the internal medicine specialists and radiologists, no clear etiology for the SOVT was identified and a diagnosis of spontaneous or incidental SOVT was made. She was commenced on intravenous ceftriaxone sodium 1 g daily, topical ocular lubricants, three classes of topical IOP lowering medications (brimonidine, dorzolamide, and timolol), oral prednisolone 60mg daily, and anticoagulation with enoxaparin (low molecular weight heparin) 60 units subcutaneously. 

Her symptoms gradually improved, until she achieved full resolution on Day 12 of admission. Her visual acuity improved to 20/40 in each eye, she had full restoration of extraocular movement and resolution of proptosis. The right eye IOP returned to normal at 14mmHg. She was discharged, tapered off the steroid regimen, and anticoagulant therapy was switched to oral warfarin, with planned follow-up in the outpatient internal medicine clinic.

Each of the cases discussed above revealed a separate etiology for SOVT. The clinical course of the cases is further summarized in Table 1 below. 

**Table 1:** Summary of the clinical course of three patients presenting with SOVT to a single hospital ophthalmology department in Trinidad and Tobago. ENT: ear, nose, throat (otolaryngology); SOVT: superior ophthalmic vein thrombosis; TED: thyroid eye disease; IOID: idiopathic orbital inflammatory disease; CCF: carotid-cavernous fistula; DM: diabetes mellitus; HTN: hypertension; IV: intravenous; SC: subcutaneous; IOP: intraocular pressure

Case #	Gender	Age	Comorbidities	Laterality	History	Presenting Signs	Differential Diagnoses	Radiological Findings	Diagnosis; Etiology of SOVT	Treatment	Outcome
1	M	44	DM	Left	2-day history of facial swelling. Diagnosed and managed as paranasal sinusitis and preseptal cellulitis by ENT team. Evolution and worsening of symptoms, with orbitopathy while admitted.	Facial swelling, evolving on Day 4 admission with lid edema, chemosis, proptosis, ophthalmoplegia, blurry vision	Facial cellulitis, orbital cellulitis, cavernous sinus thrombosis	Initial CT scan: paranasal sinusitis and facial cellulitis. Repeat CT scan and MRV: paranasal sinusitis, facial cellulitis, periorbital cellulitis, left SOVT	Paranasal sinusitis (septic)	IV antibiotics, IV steroids, SC anticoagulants	Full resolution. To continue anticoagulant therapy over 3 months
2	F	46	Nil	Left	2-week history of painful swollen eye	Periorbital edema, erythema, proptosis, chemosis, dilated tortuous conjunctival vessels	TED, IOID	CT, MRV: thickening of medial and lateral recti muscle bellies with sparing of the anterior tendons; left SOVT	TED (aseptic)	Oral steroids	Full resolution. Follow up with endocrinology.
3	F	83	DM, HTN	Right	4-week history of gradual pain and swelling of the eye	Lid edema, chemosis, proptosis, ophthalmoplegia, blurry vision.	Orbital cellulitis, TED, CCF, IOID	CT: Right-sided proptosis, right SOVT	Incidental	IV antibiotics, topical IOP medications, topical lubricants, oral steroids, SC then oral anticoagulants	Full resolution. To continue anticoagulant therapy over 3 months

## Discussion

SOVT is a classically rare but serious condition that demands prompt intervention. If not properly diagnosed and treated, any delay in management can result in permanent visual loss and even fatality [1,3]. To our knowledge, this is the first documented case series of SOVT in Trinidad and Tobago and the wider Caribbean and Latin American region.

Analysis of the factors across all three cases demonstrated no unifying etiology for SOVT, suggesting that each case was unique and the timing was purely coincidental. Recent literature reviews by Sotoudeh et al. [1], Lu Chen et al. [3] of the United States, and van der Poel et al. of the Netherlands [2], have not demonstrated any significant associations between the onset of SOVT and age, ethnicity, gender or geographic location.

SOVT was found to be a unilateral disease in all of our cases. This is consistent with the literature review, which showed that 85% of cases were unilateral [2]. In this case series, two of the three cases (66.7%) were aseptic, which also reflects the rate found in the existing literature [2]. The most common cause of septic SOVT was found to be paranasal sinusitis [2] which corresponds to the etiology of Case 1. 

Recent case reports have suggested an association between SOVT and infection with the SARS-CoV-2 virus and the ChAdOx1-S COVID-19 vaccine [5-12]. Neither SARS-CoV-2 infection nor vaccination appeared to be associated with the development of SOVT in any of these reported cases. Case 1 reported Sars CoV-2 infection in 2020 and 2021. Cases 2 and 3 reported no known infection with the virus up to the time of presentation with SOVT. Cases 1 and 2 were vaccinated in 2020 with the BBIBP-CorV COVID-19 vaccine (Sinopharm) while Case 3 was unvaccinated. This led us to conclude that the presentations of these cases were independent of either acute infection with COVID-19 or the vaccine.

Anatomy

A brief recall of the anatomy of the SOV is important in comprehending the pathophysiology of SOVT and its complications. The SOV originates from the junction of the supraorbital and angular veins at the medial margin of the supraorbital rim. It then travels posteriorly and laterally along the superolateral border of the optic nerve. As the SOV continues posteriorly, it anastomoses with the inferior ophthalmic vein and then courses through the superior orbital fissure, ultimately draining into the cavernous sinus [14].

The valveless nature of the SOV makes it more prone to occlusions [13] and can also allow for anterior infections to easily spread posteriorly [15]. Further posterior spread into the cavernous sinus can lead to the formation of cavernous sinus thrombosis (CST) or meningitis [3, 15].

Etiology

The etiology of SOVT is multifactorial with risk factors including at least one trigger from Virchow’s triad: vascular damage, stasis, and hypercoagulability [2, 4, 16]. SOVT can be broadly classified into four (4) main etiologies: septic (paranasal sinusitis, orbital cellulitis); aseptic (systemic inflammatory autoimmune diseases, coagulopathy, carotid-cavernous fistula, neoplasia), post-traumatic, and incidental [1].

Clinical presentation

SOVT symptoms are secondary to impaired orbital venous drainage including orbital swelling, pain, chemosis, eyelid edema, proptosis, limited ocular motility, with or without fundus findings, and impaired visual acuity [2]. The presentation of SOVT varies widely between cases, presenting a diagnostic challenge. Ruling out other potentially serious differential diagnoses such as orbital cellulitis and cavernous sinus thrombosis is essential. 

In two of the three cases (Cases 2 and 3) disc edema and scattered flame hemorrhages were observed. These findings were a direct result of impaired orbital venous drainage, which leads to stasis and volumetric changes in the orbital content. An orbital compartment syndrome develops, obstructing the pial circulation and subsequently leading to ischemia of the optic nerve [1,13].

Diagnosis

Proper history-taking and clinical examination are important in the patient’s initial presentation to arrive at a list of differential diagnoses. History-taking should focus on the onset of symptoms, recent or current infections, recent surgeries, medications, malignant diseases, and other systemic conditions including autoimmune disease [13].

Due to the overlap in clinical signs and range of possible differential diagnoses, radiological imaging using contrast-enhanced CT or MRI plays a critical role in the diagnosis of SOVT [1, 2]. Dilatation of the SOV with an intraluminal filling defect is the pathognomonic sign of SOVT [1, 15]. Associated radiological findings include soft tissue fat stranding around the SOV and within the orbital fat and enlargement of the extraocular muscles. It is important to note that asymmetric dilation of the SOV is not diagnostic for SOVT, as only approximately 10% of dilated SOV is due to thrombosis [1, 17].

In this case series, the images were reviewed by a senior general radiologist. Filling defects in the dilated SOV were considered diagnostic and conclusive. For each case, all available images were reviewed, documenting orbital complications of SOVT. Although not found in these cases, further analysis of the radiographs was undertaken to determine if there was an extension of the thrombosis to the cavernous sinuses and intracranial structures.

From a radiological and diagnostic perspective, early image acquisition and diagnosis are usually achieved via CT scan. Intravenous contrast is usually not necessary, as the differing densities of orbital fat and muscle allow for adequate delineation of the orbital contents. In this series, the thrombus was identified solely on CT with contrast administration in Case 3 (Figure 4). Cases 1 and 2 required MRI imaging for confirmation (Figures 2 and 3).

A venous obstruction at a level higher than the central retinal vein may result in patients presenting with high intraocular pressure, corneal edema, and iris vessel congestion. These signs should prompt immediate imaging studies to investigate possible obstructions at a more distal level [3]. MR venography or CT angiography confirms the diagnosis of isolated SOVT by ruling out shunting lesions such as carotid cavernous fistula (CCF), thrombus extension from the cavernous sinus, and sino‐orbital infections [1,3]. Additional radiological sequences such as diffusion‐weighted imaging may also be useful to highlight abnormal vasculature where contrast medium administration is contraindicated [4].

Aseptic SOVT is associated with systemic inflammatory diseases such as Graves’ disease, systemic lupus erythematosus, vasculitis, and ulcerative colitis, among others [1,13]. We detected SOVT secondary to thyroid-suspected orbitopathy, as seen in Case 2. Although unilateral in presentation with normal TFTs, the radiological findings in this patient were pathognomonic for the typical appearance of thyroid-associated orbitopathy: sparing of the anterior tendons with swelling confined to the muscle bellies [18]. A recent literature review of the asymmetric presentation of Grave’s orbitopathy estimated the prevalence of unilateral orbitopathy to be between 4.5-14%, while asymmetrical orbitopathy was reported to be 9-34%, the cause of which is still yet undefined [19]. In patients who develop TED, 85-90% are found to be hyperthyroid, 5-6% euthyroid, and 4% are hypothyroid [18]. 

A brief summary of other critical differential diagnoses of SOVT, along with their key clinical features, are shown in Table 2 below [20,21] to review the diagnostic process. 

**Table 2:** Brief summary of differential diagnoses of superior ophthalmic vein thrombosis

Differential Diagnosis	Etiology	Clinical Features	Associated Conditions
Orbital cellulitis	Septic causes	Swollen, erythematous and tender lids; chemosis; discharge; proptosis; optic neuropathy; pyrexia	Sinus infections; mid-facial skin infection; recent dental surgery; trauma
Mucormycosis		Swollen, erythematous, and tender lids; chemosis; discharge; proptosis; optic neuropathy; pyrexia; black eschar of skin and turbinates	Diabetic ketoacidosis; immunocompromised
Thyroid eye disease	Aseptic causes	Lid retraction; proptosis; restrictive myopathy; optic neuropathy	Hyper/hypothyroidism, Grave's disease, Hashimoto's thyroiditis
Sarcoidosis		Lid and conjunctival granulomas; uveitis; proptosis; ophthalmoplegia; optic neuropathy	Systemic inflammatory disease. Non-caseating granulomatous inflammation on histology
Idiopathic orbital inflammation		Proptosis; chemosis; painful ophthalmoplegia; optic neuropathy	No known etiology. Nonspecific, polymorphous inflammatory infiltrate on histology.
Cavernous sinus thrombosis	Vascular causes	Headache; nausea and vomiting; proptosis; chemosis; congestion of conjunctival vessels; third to sixth cranial nerves palsy	Sinusitis; orbital or preseptal cellulitis
Carotid cavernous fistula		Direct: pulsatile proptosis; conjunctival chemosis and vessel engorgement; bruit; high intraocular pressures. indirect: gradual onset and milder features; conjunctival vessel engorgement; high intraocular pressures; pulsatile proptosis	Prior trauma or surgery; atherosclerosis
Cavernous hemangioma		Proptosis; ophthalmoplegia; optic neuropathy	Maffucci syndrome
Orbital lymphoma	Tumors and neoplasia	Proptosis; ophthalmoplegia; periorbital swelling; pain; optic neuropathy; chemosis; palpable orbital mass	Non-Hodgkin lymphoma
Lacrimal gland lesions		Inferomedial proptosis; ptosis; palpable mass	Tumors, inflammatory processes, and infiltrative processes
Optic nerve glioma and meningioma		Proptosis; ophthalmoplegia; optic disc swelling; optic neuropathy; disc collaterals	Neurofibromatosis type 1; neurofibromatosis type 2
Metastatic lesions		Proptosis; pain; diplopia; ptosis	Breast carcinoma, lung carcinoma, prostate carcinoma, cutaneous melanoma

Management

At present, only recommendations, as opposed to established guidelines, exist for the management of SOVT [13]. In cases of septic causes of SOVT, empirical treatment with broad-spectrum intravenous antibiotics should be commenced initially, pending culture results [13]. This regime was implemented in Case 1. Consideration should be given to agents covering* Staphylococcus aureus* (including methicillin-resistant *Staphylococcus aureus*) as these were the most common causative bacteria [3]. 

Aseptic causes of SOVT, as well as post-traumatic and incidental causes, can be managed by a combination of anticoagulant and steroid therapy [1]. Anticoagulants can reduce the risk of thrombosis progressing toward the cavernous sinus which can lead to a fatal outcome. Heparin, either dose-adjusted intravenous heparin or low molecular weight heparin, is the initial anticoagulant of choice [3, 13] and was used in Cases 1 and 3 (enoxaparin).

Existing evidence shows that the most commonly prescribed steroid for cases of SOVT is intravenous dexamethasone [3]. Although the three cases in this review were commenced on steroids, only Case 1 was prescribed intravenous dexamethasone; Cases 2 and 3 were prescribed oral prednisolone. There was no obvious difference in the clinical outcomes based on either the choice or mode of delivery of steroid treatment.

When medical management is inadequate to treat the underlying cause or in the presence of orbital abscess, sinus disease, or risk of orbital compartment syndrome, urgent surgical intervention is indicated [13, 14]. Critically, the management of SOVT should be individually tailored depending on the etiology [1].

Limitations

This paper is a description of three cases presented as a case series. An inherent limitation is the small number of cases and lack of comparative data for Trinidad and Tobago, the wider Caribbean, and the Latin American region. 

Resources required for certain diagnostic laboratory tests were unavailable and cost-prohibitive to our patients, such as thyroid auto-antibodies in Case 2.

No standardized follow-up imaging was performed on any of the three cases to confirm radiologic resolution of SOVT or evidence of reversal of SOV dilatation. We therefore relied on clinical features to determine the resolution of SOVT symptoms. 

## Conclusions

This paper introduces three new cases of superior ophthalmic vein thrombosis (SOVT) to the existing and sparse body of literature. These cases underscore the heterogeneity of SOVT, the importance of radiological investigations, and the need for individualized management approaches, tailored to the underlying etiology and patient-specific factors. A multidisciplinary approach involving ophthalmologists, radiologists, internal medicine specialists, and hematologists is crucial. Complete clinical resolution was achieved in all three cases after collaborative management which included a combination of intravenous antibiotics, oral and intravenous steroids, and anticoagulation using heparin. 

Consideration should be given to future studies to further explore the use of non-contrast-enhanced imaging in the investigation and management of SOVT. These may include clinically correlated reviews of contrast-enhanced CT or MR, diffusion‐weighted imaging, and non-contrast investigations, such as Doppler ultrasound and OCT for monitoring optic nerve and retinal changes in SOVT patients. These investigations may facilitate faster and more efficient diagnoses and provide an option for those in whom contrast is contraindicated.

Due to the paucity of evidence for this condition, we recommend robust reporting of SOVT to the existing literature via case reports and series, the result of which can form the basis of future systematic reviews, meta-analyses, and ultimately, the establishment of clearer management protocols for SOVT. Possible novel causes for the development of SOVT such as COVID-19 infection and the COVID vaccine, should be considered with each new presentation of SOVT for which a clear etiology is undefined. 
